# Shifts in intestinal microbiota and improvement of sheep immune response to resist Salmonella infection using Toll-like receptor 4 (TLR4) overexpression

**DOI:** 10.3389/fmicb.2023.1075164

**Published:** 2023-02-15

**Authors:** Xue-Ling Xu, Yue Zhao, Ming-Ming Chen, Yan Li, Yao Li, Su-Jun Wu, Jin-Long Zhang, Xiao-Sheng Zhang, Kun Yu, Zheng-Xing Lian

**Affiliations:** ^1^Beijing Key Laboratory for Animal Genetic Improvement, National Engineering Laboratory for Animal Breeding, Key Laboratory of Animal Genetics and Breeding of the Ministry of Agriculture, College of Animal Science and Technology, China Agricultural University, Beijing, China; ^2^Institute of Animal Husbandry and Veterinary Medicine, Tianjin Institute of Animal Sciences, Tianjin, China

**Keywords:** TLR4, intestinal microbiota, 16S rRNA, inflammation, immune

## Abstract

**Introduction:**

Toll-like receptor 4 (TLR4) identifies Gram-negative bacteria or their products and plays a crucial role in host defense against invading pathogens. In the intestine, TLR4 recognizes bacterial ligands and interacts with the immune system. Although TLR4 signaling is a vital component of the innate immune system, the influence of TLR4 overexpression on innate immune response and its impact on the composition of the intestinal microbiota is unknown.

**Methods:**

Here, we obtained macrophages from sheep peripheral blood to examine phagocytosis and clearance of Salmonella Typhimurium (*S. Typhimurium*) in macrophages. Meanwhile, we characterized the complex microbiota inhabiting the stools of TLR4 transgenic (TG) sheep and wild-type (WT) sheep using 16S ribosomal RNA (rRNA) deep sequencing.

**Results:**

The results showed that TLR4 overexpression promoted the secretion of more early cytokines by activating downstream signaling pathways after stimulation by *S. Typhimurium*. Furthermore, diversity analysis demonstrated TLR4 overexpression increased microbial community diversity and regulated the composition of intestinal microbiota. More importantly, TLR4 overexpression adjusted the gut microbiota composition and maintained intestinal health by reducing the ratio of Firmicutes/Bacteroidetes and inflammation and oxidative stress-producing bacteria (Ruminococcaceae, Christensenellaceae) and upregulating the abundance of Bacteroidetes population and short-chain fatty acid (SCFA)-producing bacteria, including Prevotellaceae. These dominant bacterial genera changed by TLR4 overexpression revealed a close correlation with the metabolic pathways of TG sheep.

**Discussion:**

Taken together, our findings suggested that TLR4 overexpression can counteract *S. Typhimurium* invasion as well as resist intestinal inflammation in sheep by regulating intestinal microbiota composition and enhancing anti-inflammatory metabolites.

## 1. Introduction

*Salmonella Typhimurium* (*S. Typhimurium*), a zoonotic pathogen causing public health hazards, is endemic in many countries, where it causes significant economic losses due to the gastrointestinal infections in ovine hosts (Alvseike and Skjerve, 2002). Outbreaks of *S. Typhimurium* in sheep flock is characterized by rapid spread and severe mortality in ewes and lambs. Sheep are hosts for the pathogens of latent zoonoses, and larger Salmonella outbreaks have been reported in association with the consumption of sheep meat (Carson and Davies, 2018). Recurrent epidemics of salmonellosis in New Zealand associated with exposure to sheep (Baker et al., 2007). Toll-like receptors (TLRs), a family of pattern-recognition receptors expressed mainly in innate immune cells, are essential to initiating inflammatory responses as well as early immune defenses by detecting pathogen-associated molecular patterns (PAMPs) from a variety of pathogens (Kawai and Akira, 2007). During infection, TLR4 specifically recognizes endotoxin lipopolysaccharide (LPS), a primary component of Gram-negative bacteria, which induces immune cells such as macrophages and neutrophils to produce many pro-inflammatory cytokines, including interleukin (IL)-8, IL-6, IL-1β and IL-12 and tumor necrosis factor α (TNFα), to eliminate the invading pathogens (Ramachandran, 2014). Understanding the resistance of TLR4 to *S. Typhimurium* infection will help reduce clinical disease in animals and decrease the risk of foodborne transmission.

Importantly, TLRs recognize not only pathogens, but also commensal bacteria, and this interaction is crucial to maintain homeostasis in the intestinal epithelium cells under normal physiological conditions. The intestinal bacteria are a very complex and diverse ecosystem consisting of millions of microorganisms that inhabit the mammalian gastrointestinal tract. The phyla Firmicutes (Gram-positive) and Bacteroidetes (Gram-negative) are the dominant phyla of sheep gut microorganisms that produce various metabolites capable of influencing the physiology of the host (Zhang et al., 2022). Whereas bacteria belonging to the Proteobacteria, Verrucomicrobia, or Actinobacteria phyla are usually minor constituents. Ruminococcaceae is the main microorganisms in the intestinal tract of sheep and is related to energy metabolism and has an important role in the degradation of cellulose and starch (Sun et al., 2019).

Intestinal bacteria not only promote nutrients absorption by secreting enzymes to break down other indigestible substances, but they are also involved in inflammation, immune system development, and the gut-brain axis regulation (Hill and Artis, 2010; Lozupone et al., 2012; Karakan et al., 2021). Disruption of microbiota structure is related to diseases, including obesity, malnutrition, autoimmunity, inflammatory bowel disease (IBD), and infectious diseases (Donskey et al., 2000; Ley et al., 2005; Frank et al., 2007; Turnbaugh et al., 2008; Wu et al., 2010; Elinav et al., 2011; Kau et al., 2011). Among these, the presence of commensal bacteria in the gut seems to be crucial for the pathogenesis of IBD, including Crohn’s disease and ulcerative colitis. Chronic inflammation of the gut is usually characteristic of these diseases and is considered to be due to abnormal activation of the immune system by bacteria (Farrell and LaMont, 2002; Mirsepasi-Lauridsen et al., 2019). In healthy conditions, the intestinal mucosal barrier separates the gut bacteria from the rest of the host and consists of epithelial cells and proteins that participate in mucosal homeostasis (Okumura and Takeda, 2017). Bacterial products linking TLRs to intestinal epithelial cells can promote epithelial cell proliferation, IgA secretion and antimicrobial peptides expression (Abreu, 2010; Venkatesh et al., 2014). The presence of IgA decreases intestinal pro-inflammatory signaling in the host (Visekruna et al., 2019). Therefore, the immune system also affects the intestinal bacteria composition. The segmented filamentous bacteria (SFB) expanded aberrantly in the gut of mice deficient in IgA (Suzuki et al., 2004). Deficiency of NLRP6 in mouse colonic epithelial cells results in altered fecal microbiota characterized by increased colitis in NLRP6 KO mice and wild-type (WT) mice (Elinav et al., 2011). Alterations in the composition of the gut microbiota have also been associated with TLR signaling. The cecal microbiota of mice genetically deficient in TLR5, for instance, different from WT littermates in 116 bacterial phylotypes from diverse phyla (Vijay-Kumar et al., 2010). The distal gut bacterial composition was altered in MyD88-deficient mice, with higher abundance of the Rikenellaceae and Porphyromonadaceae bacterial families in cecal microbiota (Wen et al., 2008).

Given that the immune system has an impact on the gut microbiota, in this study, we were concerned about the role of TLR4 signaling in defense against Salmonella *S. Typhimurium* (a food borne pathogen) invasion and on the intestinal microbiota composition of sheep. We analyzed the microbiota of TLR4 overexpressing sheep and their WT controls using high-throughput sequencing of 16S rRNA genes amplified from fecal samples. An objective of the current study was to determine whether TLR4-overexpressing alters the composition of the intestinal microbiota in sheep.

## 2. Materials and methods

### 2.1. Ethics statement

All sheep were housed in accordance with the national feeding standard NT/T815--2004. All procedures performed for this study were consistent with the National Research Council Guide for the Care and Use of Laboratory Animals. All experimental animal protocols in this study were approved and performed in accordance with the requirements of the Animal Care and Use Committee at China Agricultural University (approval number AW81012202-1-3).

### 2.2. Animals

**Figure 1:**
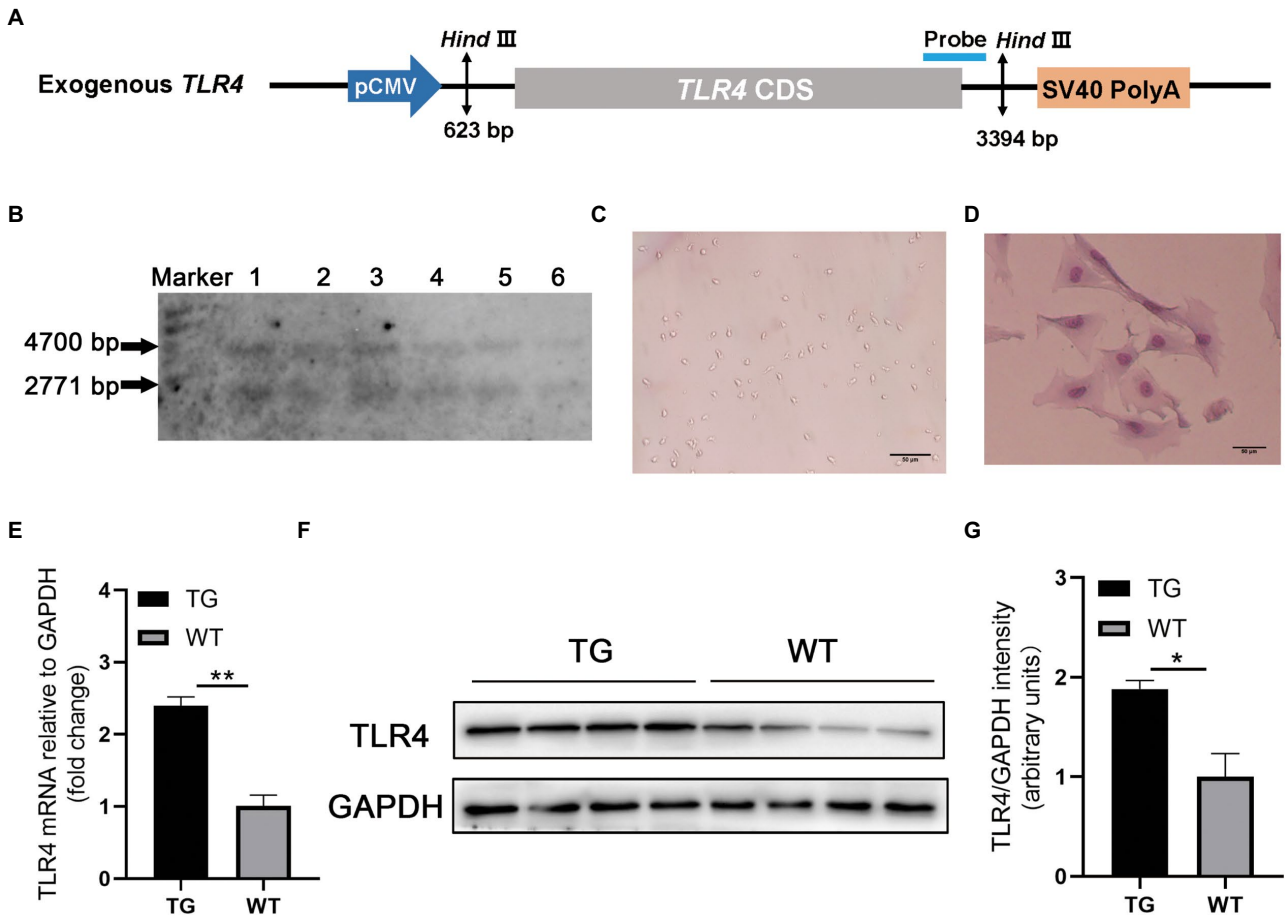
TLR4 overexpression assay in sheep. **(A)** Structure diagram of CMV-ovis aries TLR4 overexpression vector. **(B)** Identification of TG sheep by representative southern blot based on the presence of the TLR4 transgene. TG sheep have both an endogenous 4,700 bp TLR4 band and an exogenous 2,771 bp TLR4 band. Marker, 1 kb ladder. **(C)** Morphology of isolated macrophages. **(D)** Sheep macrophages were isolated from peripheral blood and their morphology was highlighted by Giemsa staining. **(E)** The mRNA expression of TLR4 in macrophages was examined by qRT-PCR. **(F)** TLR4 protein expression in macrophages was analyzed using western blotting. Compared with WT macrophages, the TLR4 protein level in TG macrophages was significantly higher. **(G)** The data of western blot were analyzed statistically. TG, transgenic; WT, wild-type. Error bars indicate SD, *N* = 4; *p < 0.05, **p < 0.01.

The linear vector including *ovis aries* TLR4 (Figure 1A) was microinjected into the fertilized egg to obtain founder transgenic (TG) sheep (Deng et al., 2012). The founder TG sheep were bred with WT sheep to breed offspring. In the offspring, healthy female sheep aged 2 ∼ 3 years were identified as WT or TG by Southern blotting (Roche Diagnostics, Germany) (Bai et al., 2015).

20 μg genomic DNA from ear tissue was extracted and digested by HindIII (New England Biolabs, Britain). Designed Southern blotting probes and prepared PCR products. The primer used are following: forward 5′-ACTGGTAAAGAACTTGGAGGAGG-3′ and reverse 5′-CCTTCACAGCATTCAACAGACC-3′, and the 671-bp PCR product labeled with digoxigenin (Roche Diagnostics, Germany). There were five *Ovis aries* in the TG group and five *Ovis aries* in WT group for the subsequent experiments. In this study, all TLR4 overexpressing and WT female sheep were provided by the Institute of Animal Science and Veterinary Medicine Tianjin Academy of Agricultural Sciences and kept in the same environment.

### 2.3. Isolation and culture of macrophages

The peripheral blood (10 mL) of sheep was collected from the jugular vein, and peripheral blood lymphocyte separation solution (TBDscience, China) was added promptly. After density gradient centrifugation (2,000 rpm for 30 min at 4°C), the peripheral blood mononuclear cells were washed with PBS. Mononuclear cells were resuspended in RPMI 1640 medium (Gibco, USA) supplemented with 10% fetal bovine serum (FBS, Gibco) and 1% penicillin–streptomycin, then cultured in 6-well culture plates at 37°C with 5% CO2. After 2 h of incubation, the wells were washed with PBS to remove non-adherent cells, and single adherent mononuclear cell was differentiated into spindle-shaped macrophages, giving rise to proliferating clones after 1 week (Figure 1C).

### 2.4. Macrophages infection and colony-forming unit counts

The *S. Typhimurium* was preserved in our laboratory. The bacterial strain was cultured in Luria broth (LB) medium at 37°C to the logarithmic growth phase for later using. To detect the phagocytic capability of the TG and WT macrophages, cells were infected with live *S. Typhimurium* CVCV541. The bacteria were suspended in RPMI-1640 medium (Thermo Fisher Scientific, USA) with 10% FBS. Then the adherent macrophages were infected with *S. Typhimurium* at two different levels of MOI (5 and 10) for 5, 15, and 30 min, and were washed with PBS including 100 μg/ml gentamicin. Then the cells were lysed by 0.25% tritonX-100 to release intracellular bacteria and the number of intracellular survival bacteria was quantified by counting CFUs after serial dilution.

In order to determine the bacterial clearance capacity in macrophages, macrophages were first infected with *S. Typhimurium* CVCV541 for 30 min. Extracellular bacteria were removed by washing the macrophages with PBS including 100 μg/mL gentamicin for three times. Then add RPMI-1640 containing 10% FBS to the cells for 30 min, which was recorded as the time point 60 min. The cells were washed and lysed with 0.25% Triton X-100 (in PBS). Calculate the phagocytosis time from the time of adding bacteria. The data on the number of cleared bacteria were accessed by subtracting intracellular bacteria number in 60 min from the bacteria number in 30 min.

### 2.5. Quantitative real-time PCR (qRT-PCR)

In order to compare the expression of factors between the TG and WT groups in sheep under the attack of *S. Typhimurium*, the cells were treated with *S. Typhimurium* SL1344 for 0, 0.5, and 4 h at a MOI of 10, and the mRNA expression of related genes was detected and quantified by RT-PCR. Total RNA from sheep macrophages was extracted using RNA extraction kit (Aidlab, Beijing, China) according to the manufacturer’s instructions. RNA was treated with DNAse I to remove any residual DNA, and the quantity and quality of RNA were assessed with a NanoDrop 2000 spectrophotometer (Thermo Scientific, Waltham, MA). The extracted 1 μg of RNA was reverse transcribed into cDNA using PrimeScript RT kit (TaKaRa). The cDNA was analyzed by RT-qPCR. The gene primers shown in Table 1 were synthesized from tsingke (Beijing, China). qRT-PCR was performed using the SYBR Premix Ex Taq II kit (TaKaRa, Kyoto, Japan). Mx3000P instrument (Agilent Technologies, USA) was used to acquire RT quantitative PCR data. The qPCR equipment automatically calculates the cycle number of PCR product fluorescence above the background signal, referred to as the cycle threshold (Ct). The data were analyzed using the comparative Ct technique (2^−ΔΔCT^ method) by averaging the Ct values from three replicates, and the expression levels of mRNA were normalized to housekeeping gene GAPDH mRNA levels.

**Table 1:** The primer sequences for quantitative polymerase chain reaction.

Gene	Forward (5′-3′)	Reverse (5′-3′)
IL-6	CTGCTGGTCTTCTGGAGTATC	TGTGGCTGGAGTGGTTATTAG
IL-1β	CAGGCAGTGTCGGTCATCGT	GCATCACACAGAAGCTCATCGG
IL-8	GCTGGCTGTTGCTCTCTTGG	GGGTGGAAAGGTGTGGAATGTG
TNFα	TCTACTCGCAGGTCCTCTTC	TCGGCATAGTCCAGGTATTC

### 2.6. Western blot

Cell proteins were extracted with RIPA lysis buffer (Beyotime, Beijing, China). The protein concentrations were determined by bicinchoninic acid (BCA) protein assay kit (Cwbio, Jiangsu, China), and then boiled at 95°C with 6 × loading buffer for 8 min. Samples were separated by 10% sodium dodecyl sulfate-polyacrylamide gel electrophoresis (SDS-PAGE) and immediately transferred onto 0.22 μm polyvinylidene fluoride membranes (Millipore, USA), the membranes were blocked with Tris-buffered saline with Tween 20 (TBST) containing 5% nonfat milk at room temperature for 60 min. After washing three times, the membranes were incubated overnight at 4°C with anti-TLR4 (1:1,000 dilution, AF7017, Affinity). Then blots were bound with horseradish peroxidase (HRP)-conjugated secondary antibodies (1:20,000 dilution, Proteintech) for 60 min at 37°C. After washing again with TBST three times, experimental results were obtained by the pierce enhanced chemiluminiscent (ECL) western blotting kit (Thermo Scientific), and imaged by chemiluminescence system (BIO-RAD, USA).

### 2.7. Fecal samples collection

Ten adult female sheep were used for this work. They were divided into two groups, five TLR4 overexpressing sheep for the experimental group (TG group), along with five WT sheep for the control conditions (WT group). Fecal samples were taken from the TG and WT groups with clean plastic gloves in autumn. Fresh and individualized samples were collected from two groups of sheep. Samples are transported on dry ice and stored at −80°C for subsequent experiments.

### 2.8. DNA extraction of fecal samples

Total bacterial genomic DNA samples were extracted from 10 sheep fecal samples using the Fast DNA SPIN extraction kits (MP Biomedicals, USA), according to the manufacturer’s protocol (n = 10). The quantity and quality of isolated DNA quantity and quality were determined by a spectrophotometer (NanoDrop 2000; Thermo Scientific, USA) and agarose gel electrophoresis, respectively (Supplementary Figure S1). The extracted sample DNA was stored at −20°C until used for PCR amplification and sequencing.

### 2.9. Bacterial 16S rRNA library construction and sequencing

The prokaryotic 16S rRNA gene libraries were generated from the extracted DNA using amplicon PCR with variable V3–V4 region (Forward, ACTCCTACGGGAGGCAGCA and Reverse, GGACTACHVGGGTWTCTAAT) and the Illumina 16S Sample Preparation Guide. Preparation of 25 μL PCR components for amplification. The amplification procedure consists of an initial denaturation at 98°C for 2 min, followed by 25 cycles consisting of denaturation at 98°C for 15 s, annealing at 55°C for 30 s, extension at 72°C for 30 s, and a final extension at 72°C for 5 min, hold at 10°C. Unbound primers, other contaminants, and primer dimer fragments were removed and quantified. The purified samples were collected in equimolar amounts, and paired-end sequencing was performed with the IllluminaMiSeq platform at Shanghai Personal Biotechnology Co., Ltd. (Shanghai, China).

### 2.10. 16S rRNA gene sequencing analysis

The Quantitative Insights into Microbial Ecology (QIIME) pipeline was employed to carry out the quality control of the sequencing data (Bolyen et al., 2019). Specifically, the original sequencing reads were accurately matched to the barcodes assigned to the respective samples and determined as valid sequences. The software package DADA2 was applied (Callahan et al., 2016). When there is at least 12 bp overlap between the two reads, the reads are merged and the chimera is eliminated. The product was an amplified sequence variant (ASV) table, which has a higher-resolution and reasonable analog than the traditional operational taxonomic units (OTU) table (Callahan et al., 2017).

Sequences were classified from the phylum level to the species level. To classify and identify sequence reads using Silva version 132 as a reference database (Pruesse et al., 2007). This was achieved through the functions in the DADA2 package: assingSpecies and assingTaxonomy. The samples herein after extraction, total reads, final reads, and taxa are shown in Supplementary Table S1. ASVs from the same phylum were combined into respective groups. The default arguments of functions were applied.

### 2.11. Statistics

Statistical and bioinformatics analysis of 16S rRNA gene sequencing. The within-community diversity (α-diversity), including Chao1 richness, Shannon diversity index, and Simpson index, were detected using the ASV table in QIIME. ASV-level abundance curves were generated to compare the richness and homogeneity of ASVs in samples. Changes in community composition (β-diversity) was performed by the principal coordinates analysis (PCoA), weighted and unweighted UniFrac NMDS analysis, as well as hierarchical clustering analysis based on weighted UniFrac distances. They were performed by QIIME and R software. The Gram-positive/Gram-negative ratio was calculated by dividing the number of Actinobacteria and Firmicutes sequences by the number of Gram-negative bacteria (here defined as those phyla containing Hsp60 inserts, which are considered to most closely reflect the traditional concept of gram-negative bacteria) (Sutcliffe, 2010; Gupta, 2011). The relative abundance of Gram-positive and Gram-negative bacteria was also calculated by dividing each group by the total number of sequences found in each group. The linear discriminant analysis (LDA) and LDA effect size (LEfSe) methods were used to detect taxa of different abundant in each group. The Spearman correlation was used for correlation analysis, and the ASV table was centered and log-transformed before calculating the correlation. Use the ggplot package for visualization and plotting. Functional prediction of the intestinal microbiota was performed on Phylogenetic Investigation of Communities by Reconstruction of Unobserved States (PICRUSt 2) (Douglas et al., 2020).

All the bar and box plots were generated using GraphPad Prism version 8.0 (GraphPad Software, USA). All data were expressed as mean ± SD. One-way ANOVA was used for statistical analysis, followed by the Turkey test for multiple comparisons (*p < 0.05, **p < 0.01).

## 3. Results

### 3.1. Identification of the TLR4-overexpressing TG sheep

To confirm TLR4 expression vector (Figure 1A) is integrated into the genome of TG sheep bred in our previous research, TLR4 fragment was observed using Southern blotting. The genomes of TG and WT sheep were dissected using the restriction enzyme Hind III. TG sheep both had an endogenous TLR4 fragment of 4,700 bp and 2,771 bp exogenous TLR4 gene, whereas WT sheep had only a 4,700 bp endogenous TLR4 fragment (Figure 1B).

Monocyte-derived macrophages were obtained from sheep peripheral blood and identified by giemsa stain. The successfully isolated cells were spindle-shaped with round nuclei, some pseudopodia and vacuoles (Figure 1C). And the nuclei were darkly colored as observed by giemsa stain (Figure 1D). To further examine the TLR4 expression level, RT-PCR and Western blotting were used for evaluation, respectively. The mRNA and protein levels of TLR4 in TG macrophages were apparently higher than in WT macrophages (Figures 1E–G).

### 3.2. TLR4 overexpression increased phagocytosis and clearance of *Salmonella Typhimurium* and facilitated activation of multiple TLR4-mediated cytokines

**Figure 2:**
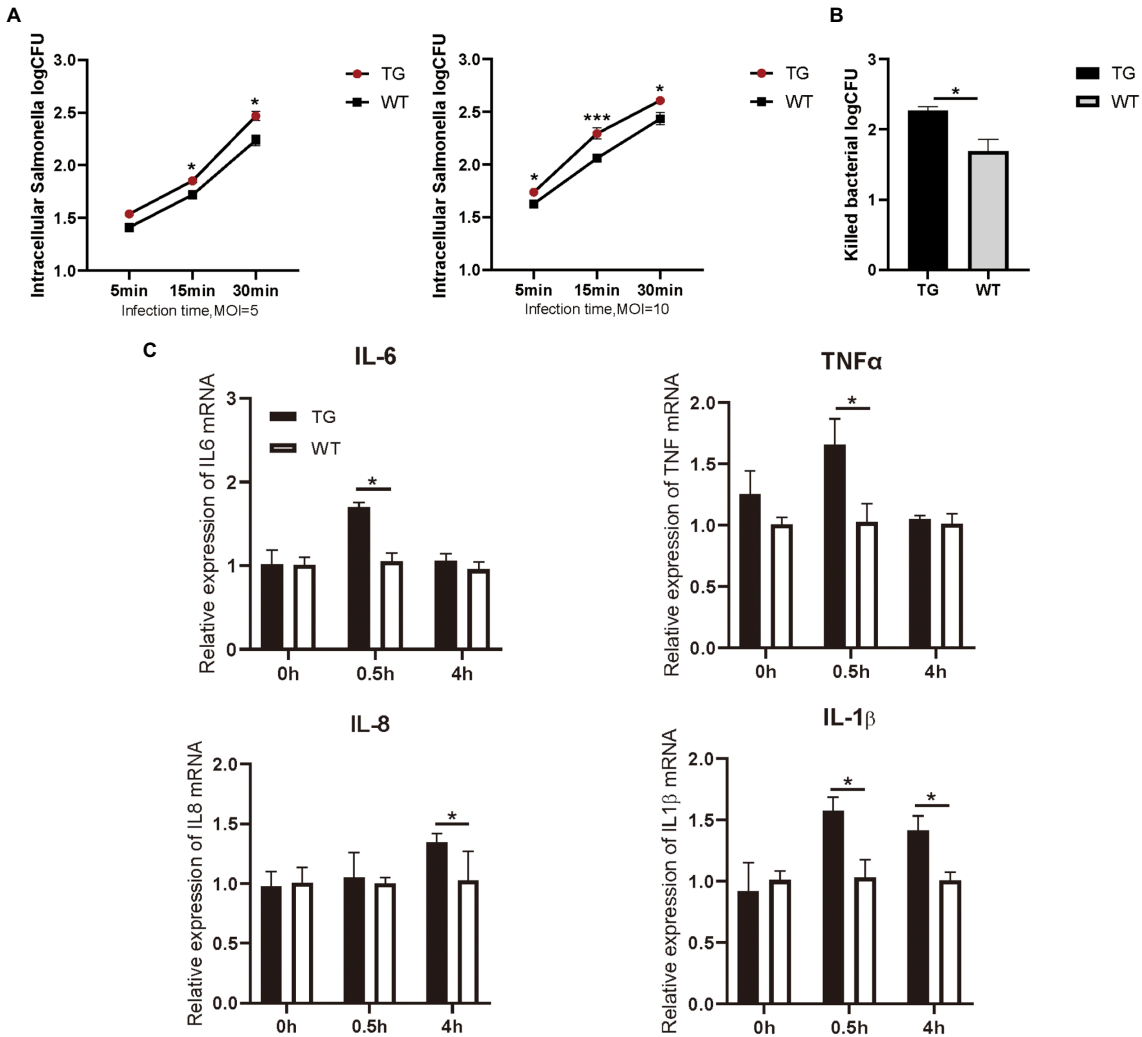
TLR4-overexpressing promotes the activation of cytokines and increases *S. Typhimurium* phagocytic uptake and killing. **(A)** Different infection times with *S. Typhimurium* (MOI = 5 or 10) and intracellular *S. Typhimurium* were detected in TG and WT macrophages. **(B)** The clearance of *S. Typhimurium* in macrophages was analyzed by CFU method. Macrophages were treated with *S. Typhimurium* (MOI = 10) for 30 min. After 30 min, survived *S. Typhimurium* in macrophages were counted by CFU plating. **(C)** The expression levels of the pro-inflammatory cytokines like IL-6, TNFα, IL-8 and IL-1β were determined in macrophages by qRT-PCR after *S. Typhimurium* for 0, 0.5, and 4 h. *N* = 4; *p < 0.05, ***p < 0.001. TG, transgenic; WT, wild-type; MOI, multiplicity of infection; CFU, colony-forming unit.

As immunocompetent cells, macrophages have a central role in body’s defense. We examined the phagocytotic capacity of TLR4 macrophages for *S. Typhimurium* CVCC541 through plate colony counting method. The amount of internalized *S. Typhimurium* in the TG and WT macrophages both enhanced with the duration of infection. However, the levels of *S. Typhimurium* in TLR4 overexpression macrophages were significantly higher (*p* < 0.05) than WT macrophages at different times after live *S. Typhimurium* treatment, regardless of the MOI 5 or 10 (Figure 2A). In other words, these TLR4 macrophages possessed a stronger engulfing capacity than that of WT macrophages. For further confirm the role of TLR4 in controlling bacterial growth, we used the CFUs assay to assess the clearance capacity of *S. Typhimurium* by TLR4 macrophages. The number of viable bacteria growing on the agar plates was determined after 16 h as CFU. Macrophages were infected for a fixed time with *S. Typhimurium* and then examined the elimination capacity of *S. Typhimurium* in TLR4 overexpressing macrophages and WT macrophages after 1 h post infection (Figure 2B). The bacterial killing capacity of *S. Typhimurium* in TLR4 macrophages was dramatically higher than that in the WT group at MOI 10 after infection with live *S. Typhimurium* (Figure 2B). These data demonstrated that TLR4 not only elevates the phagocytotic capacity of *S. Typhimurium*, but also effectively controls *S. Typhimurium* growth within macrophages.

Macrophages are critical for inducing specific immune responses and producing soluble cytokines that participate in the regulation of the generated response (Arango Duque and Descoteaux, 2014). To elucidate the effect of TLR4 overexpression on macrophage cytokine expression in the early stages of *S. Typhimurium* infection, we detected the production of IL-6, IL-1β, IL-8, and TNFα with RT-PCR. The results showed that the expression of IL-6, IL-1β, and TNFα were markedly higher (*p* < 0.05) in the TG macrophages than in the WT macrophages at 0.5 h after *S. Typhimurium* stimulation (Figure 2C). Then, at 4 h time points, the production of *S. Typhimurium*-induced IL-1β, and IL-8 in TLR4-overexpressing macrophages remained higher (Figure 2C). To evaluate responses to the TLR4 ligands were specific to their receptors, macrophages were treated with LPS (100 ng/mL) or Pam3CSK4 (1 μg/mL) and cytokine secretion was assessed. LPS treatment for 48 h significantly enhanced IL-6, IL-1β, and IL-8 expression in TG macrophages compared with WT macrophages, and treatment with TLR2 ligand remained intact, thereby indicating that the elevated cytokines are not compensated by other TLR agonists (Supplementary Figure S2). Taken together, the results suggested that the overexpression of TLR4 promoted the secretion of pro-inflammatory in the early stage of *S. Typhimurium* stimulation.

### 3.3. Effects of TLR4 overexpression on the richness and diversity of intestinal microbiome

**Figure 3:**
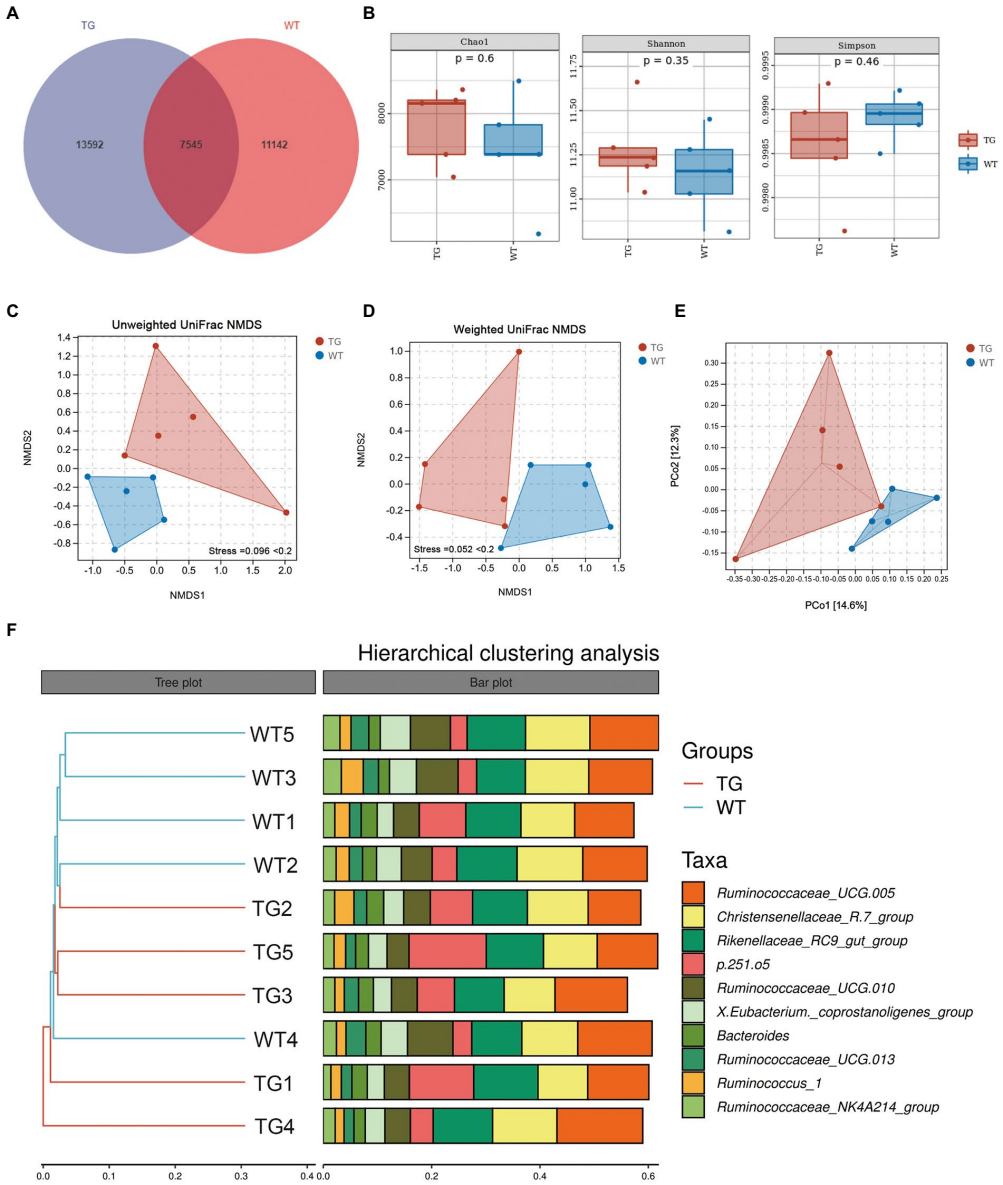
Effects of TLR4 on gut microbiota richness and diversity. **(A)** The venn diagram displayed the overlapping core microbiota of TG group and WT group. **(B)** Alpha diversity analysis was performed by Chao1 index, Shannon index and Simpson index. **(C)** NMDS analysis based on unweighted UniFrac distances. **(D)** NMDS analysis based on weighted UniFrac distances. Each point in the graph represents an individual and the shorter distance between the points, the more similar microbial community structure between individuals. **(E)** PCoA score plots of ASVs between TG and WT groups. Principal components (PCs) 1 and 2 explain 14.6% and 12.3% of the variance, respectively. The location and distance of the data points indicate the presence of bacterial taxa and the degree of similarity in relative abundance. **(F)** Hierarchical clustering analysis of ASVs by UPGMA in TG and WT groups. TG1-5: five TLR4 overexpressing sheep, WT1-5: five WT sheep.

Sheep fecal microbiota composition profiles were analyzed by the 16S rRNA gene sequencing-based method to explore the function of TLR4 in the intestinal microbiota profile. After removing unqualified sequences following previous reported method, the average number of final sequences used in the bioinformatic analysis was 59,510 per sample with a standard deviation of 11,534 (Table S1). As shown in Venn’s plot in Figure 3A, the two groups overlapped in the composition of 7,545 core microbiota. These overlapping phylotypes accounted for 35.70% (7,545/21,137) and 40.38% (7,545/18,687) of the TG group and WT group, respectively. In the study, microbial community richness and diversity were described through detecting Chao1 and Shannon indices, respectively. We observed no significant changes in Chao1 as well as Shannon indices in the TG group and WT group (Figure 3B; Supplementary Table S2). The Simpson index was also not markedly different in the two groups (Figure 3B; Supplementary Table S2). Moreover, we assessed the similarity of the global microbial community structure by evaluating β-diversity between samples, containing NMDS analysis, PCoA, as well as hierarchical clustering analysis. According to NMDS analysis based on unweighted UniFrac distances, the fecal microbiota of the TG and WT groups indicated two differentiated clusters, reflecting an alteration of fecal microbiota composition in TG sheep (Figure 3C). A similar observation can also be observed in NMDS analysis based on weighted UniFrac distances (Figure 3D).

PCoA based on unweighted UniFrac distances was used to show the effect of TLR4 overexpression on bacterial community patterns. As seen in Figure 3E, the TG and WT clustered in different coordinate spaces. Additionally, the hierarchical cluster tree constructed based on the unweighted pair group method using average linkages (UPGMA) also indicated an obvious separation between TG group and WT group (Figure 3F, WT4 individual was excluded). In conclusion, the above results denote the significant role of TLR4 in the intestinal bacterial composition.

It is worth noting that the control groups samples showed less bacterial diversity than the TG group. This low diversity could explain why the samples belonging to WT were similar according to NMDS, while individuals in the TG group were more dispersed from one another.

### 3.4. Intestinal microbiota community structure analysis

**Figure 4:**
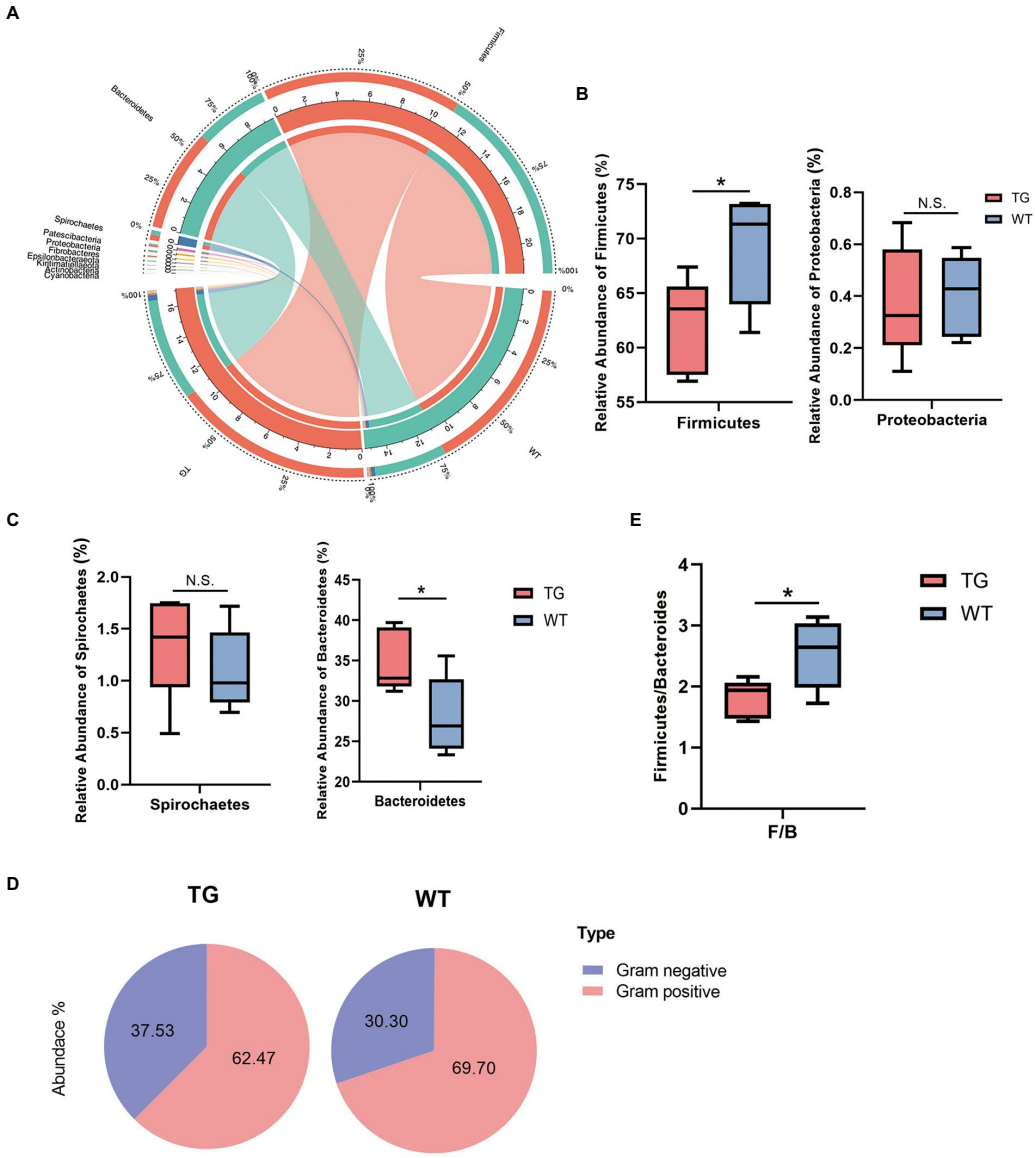
Microbiota composition of each group and changes at the phylum levels. **(A)** The correlations of microbiota with the samples in the TG group and WT group were analyzed by Circos. **(B)** Influence of TLR4 overexpression on sheep in the Firmicutes phylum and Proteobacteria phylum. The abundances of Firmicutes phylum differed significantly between TG and WT. **(C)** The relative abundance of the Spirochaetes phylum and Bacteroidetes phylum. **(D)** The pie charts depict the average abundance of Gram-positive and Gram-negative in two groups. **(E)** The Firmicutes/Bacteroidetes ratio (F/B). *N* = 5 each group; *p < 0.05 compared with WT group.

Despite this study indicated that alpha diversity was not notably affected by TLR4, the TLR4 overexpression changed the abundance of the intestinal bacteria in sheep. In practice, by using the difference analysis of the DESeq2 package, we further investigated the composition of the intestinal flora affected by TLR4 in TG vs. WT at the phylum levels. Ten different bacterial phyla were determined in two groups, which dominated in most individuals: Firmicutes, Bacteroidetes, Spirochaetes, Proteobacteria, Patescibacteria, Cyanobacteria, Fibrobacteres, Tenericutes, Actinobacteria and Verrucomicrobiota (Supplementary Figure S3). Phylum Bacteroidetes and Firmicutes significantly dominated in two groups, with the sum of the abundance of these two phyla accounting for about 95% of the total. The correlations between microbiota and individuals from the TG and WT groups were visualized by Circos analysis (Figure 4A).

On bacterial phyla abundance, Figure 4B shows the Firmicutes phylum was decreased in the TG group compared to the WT group (*p* = 0.044), which suggests the influence of TLR4 overexpression at this phylum. The Proteobacteria phylum was also decreased through TLR4 expression in TG sheep (*p* = 0.870), although not significantly compared to the WT group. Interestingly, however, overexpression of TLR4 increased the number of Spirochaetes (*p* = 0.397). The last phylum, Bacteroidetes, was the one in which TG resulted in significant population growth compared to WT (*p* = 0.039) (Figure. 4C). Supplementary Figure S4 summarized the abundance of other bacterial phyla.

Subsequently, a more detailed analysis of the proportion of Gram-positive and Gram-negative in the TG and WT was performed. The abundance of Gram-positive was decreased significantly in TG (Figure 4D), TG vs. WT (62.47 vs. 69.70) (Detailed statistical analysis is shown in Supplementary Figure S5). In addition, the Firmicutes/Bacteroidetes (F/B) ratio is widely accepted to have an important influence in regulating the intestinal immune system. We found that a reduction in the gut microbiota (F/B) ratio caused by TLR4, also reduced the number of gram-positive Firmicutes (*p* < 0.05) (Figure 4E).

**Figure 5:**
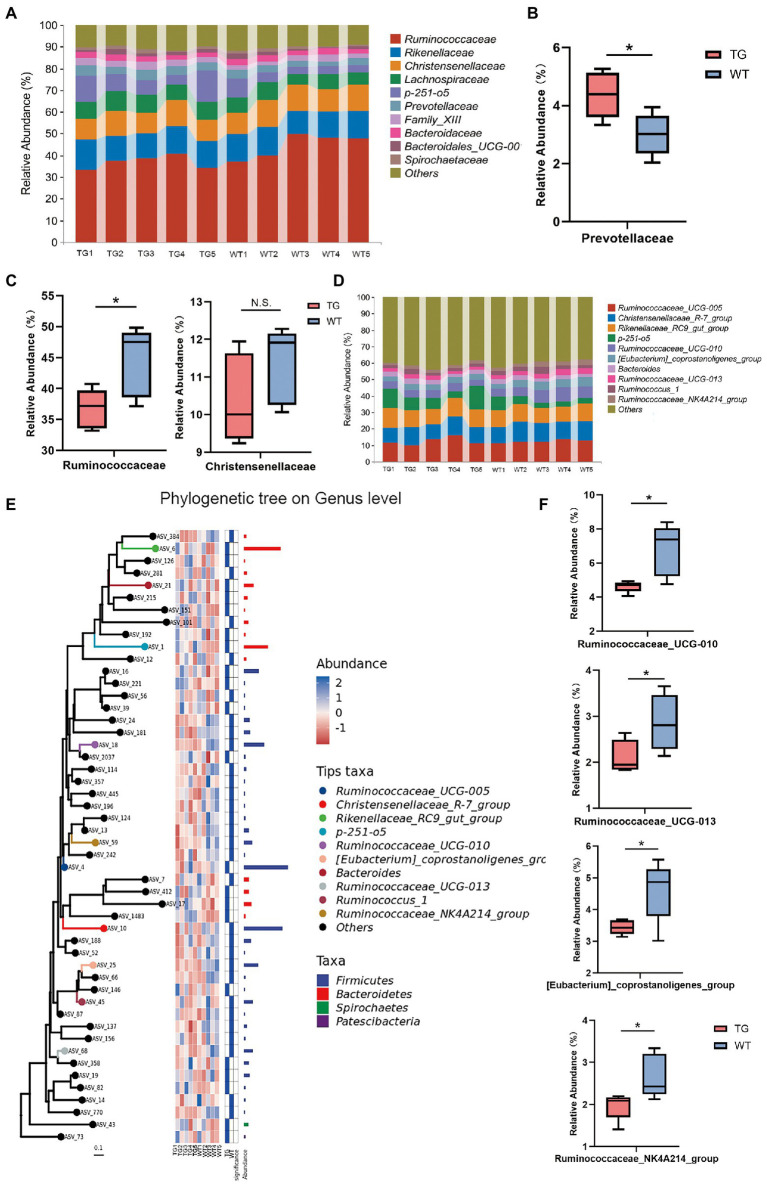
The impact of TLR4 overexpression on the composition of the intestinal microbiota in family and genus levels. **(A)** The relative abundance of fecal microbiota at the family level in all samples. **(B)** Relative abundances of Prevotellaceae. **(C)** Relative abundances of Ruminococcaceae and Christensenellaceae. **(D)** The relative abundance of fecal microbiota at the genus level. **(E)** Using phylogenetic tree to show evolutionary relationships of microorganisms at the genus level. **(F)** Box diagram of apparent changes in relative abundance at genus level. *N* = 5 each group. * *p* < 0.05 compared with the WT.

Further, the taxonomic compositions between the TG and control groups were also specifically performed at the family and genus levels (Figure 5A). At the family level, overexpression of TLR4 significantly improved the abundance of Prevotellaceae(*p* < 0.05) (Figure 5B), which metabolize short-chain fatty acids (SCFAs), enhances host intestinal barrier, and exert anti-inflammatory effects. Moreover, overexpression TLR4 reduced the abundance of Ruminococcaceae (*p* < 0.05), Christensenellaceae (Figure 5C). They produce LPS that induce inflammation and oxidative stress.

At the genus level, after filtration to remove genera showing abundance of <0.1% in the two groups, a total of 49 genera were recognized in the fecal samples, and the variation in these genera in the two groups were summarized in Supplementary Table S3. Ruminococcaceae_UCG-005, Christensenellaceae_R-7_group, Rikenellaceae_RC9_gut_group and p-251-o5 were the principal bacterial genera. Genera with relative abundance was >5%, and the composition structure of the main microbiota among aindividuals is shown in Figure 5D. The phylogenetic tree reflected the evolutionary relationships of bacteria at the genus level (Figure 5E). As seen in Figure 5F, the 10 most abundant genera in the TG group had genera that were markedly different from those in the WT group. Compared with WT group, the relative abundances of Ruminococcaceae_UCG-010, Ruminococcaceae_UCG-013, [Eubacterium]_coprostanoligenes_group and Ruminococcaceae_NK4A214_group were suppressed in the TG group (*p* < 0.05). The high expression of TLR4 also improved the abundance of Ruminococcaceae_UCG-005, Rikenellaceae_RC9_gut_group, p-251-o5, and Bacteroides (*p* > 0.05) (Supplementary Figure S6).The results of other levels are listed in Supplementary Figure S7.

### 3.5. TLR4 Overexpression altered key phylotypes of fecal microbiota

**Figure 6:**
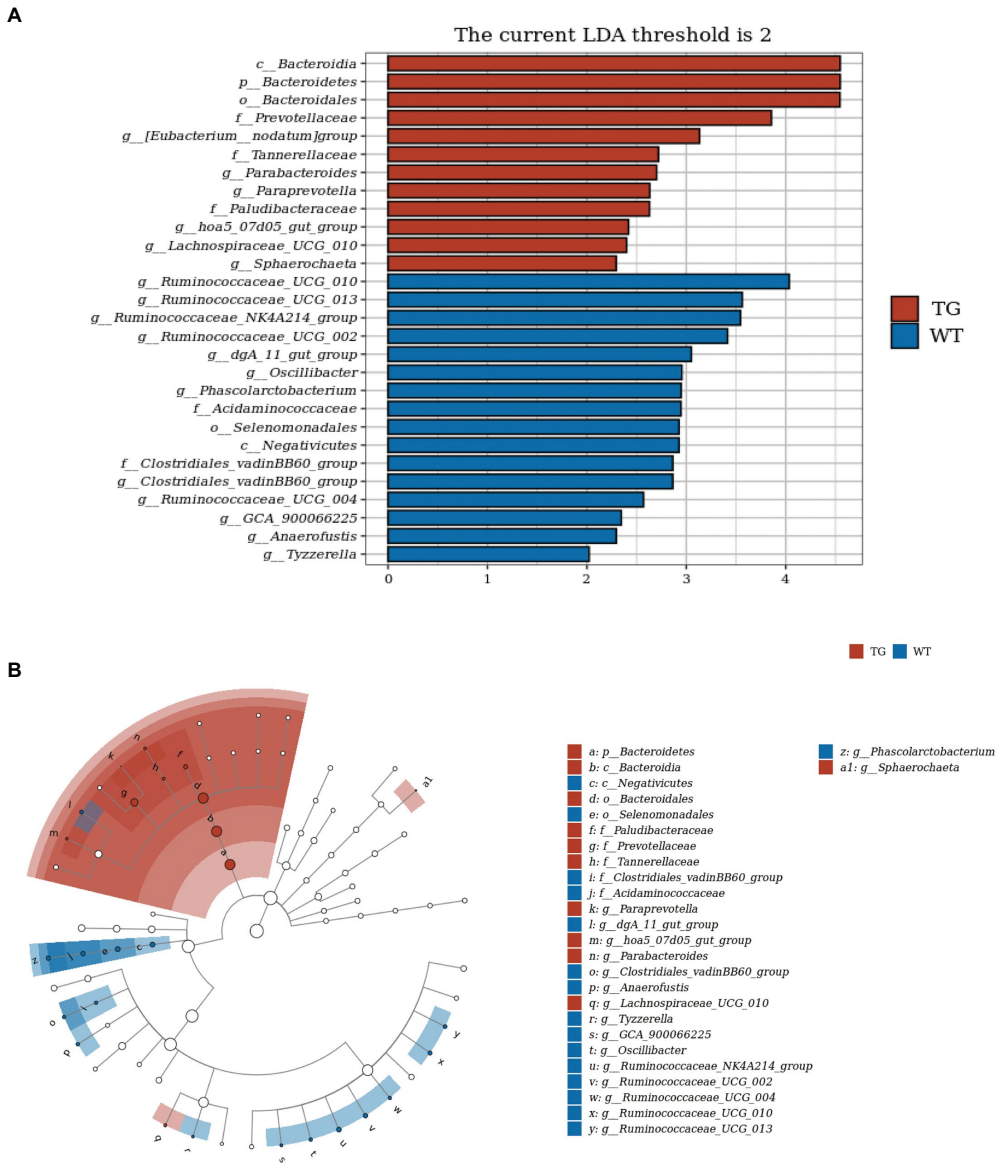
TLR4 modulated key phylotypes of gut microbiota in TG and WT. **(A)** LDA score. The enriched taxa with an LDA score > 2 is displayed in bar chart (*p* < 0.05). A larger LDA score indicates a more prominent microbiota composition. **(B)** LEfSe analysis on microbes in sheep feces on TLR4. The colored nodes from inner circle to outer circle showed the hierarchical relationship of all taxa from the phylum to the genus level. The enriched taxa in TG sheep were reported in red whereas taxa enriched in WT sheep were displayed in blue, then taxa with no significant variation were painted in white. The size of the diameter of each circle represents the taxa abundance. *N* = 5 individuals/group.

In order to visually recognize the key phylotypes of intestinal bacteria in two groups, we applied LEfSe analysis in TG sheep and WT sheep. The LDA results suggested the TG group had 12 discriminative features (LDA > 2, *p* < 0.05), with Bacteroidia, Bacteroidetes and Bacteroidales being the main microbiota. WT group also displayed 16 dominant microorganisms, and the primary microbiota were Ruminococcaceae_UGG_010, Ruminococcaceae_UGG_013, Ruminococcaceae_NK4A214_group (Figure 6A). Subsequently, an evolutionary clustering analysis diagram was delivered to identify main microflora by taxonomy (Figure 6B). In cladogram, Bacteroidetes had the highest flora abundance in the red parts and firmicutes was the richest in the blue area, which represented TG group and WT group, respectively. TLR4-induced changes in sheep microbes showed differences at the phylum and family levels, and some relative abundance changes at the genus level were also identified. In general, these results demonstrated that TLR4 expression changed the key phylotypes of gut microbiota in sheep feces and improved the proliferation of specific bacteria.

### 3.6. Role of TLR4 in metabolic pathways

**Figure. 7:**
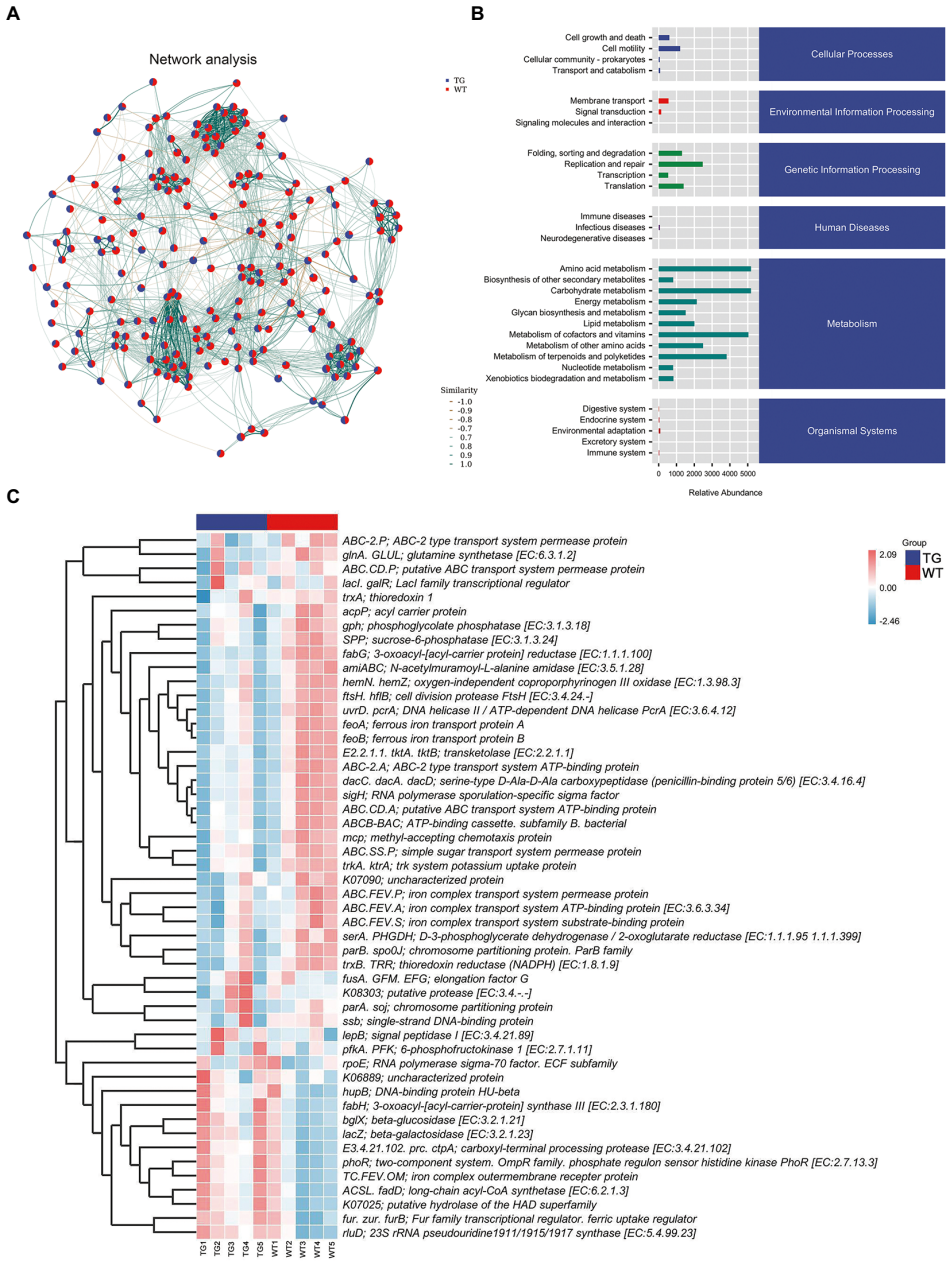
Correlation analysis of intestinal microbiota and metabolic pathways. **(A)** Network analysis. The inter-node linkage indicates an association in the two nodes being joined. **(B)** The relative abundance of metabolic pathways. **(C)** The heat map of the KEGG enriched pathways. *N* = 5 individuals/group.

Network analysis was applied at the ASV level, as displayed in Figure 7A. To determine the metabolic function of intestinal microbiota, the Kyoto Encyclopedia of Genes and Genomes (KEGG) pathways were examined using PICRUST 2. The signaling pathways were enriched in cellular processes, environmental information processing, genetic information processing, human disease, metabolism, and organismal system pathways by KEGG functional annotation analysis (Figure 7B).

Moreover, 30 level 2 KEGG orthology groups were analyzed in our samples. The results demonstrated that compared with WT, the top three increased functional abundances were the metabolism of other amino acids, glycan biosynthesis and metabolism, and energy metabolism in TG group (Figure 7B). Whereas pathways in the cell motility, transcription, signal transduction, and endocrine system were significantly decreased by TLR4 overexpression (*p* < 0.05). In the KEGG function analysis, the top three raised functional abundances were K03088 (RNA polymerase sigma-70 factor, ECF subfamily), K02529 (LacI family transcriptional regulator), K02014 (iron complex outer membrane receptor protein), whereas the top three reduced functional abundances were K01990 (BC-2 type transport system ATP-binding protein), K06147 (ATP-binding cassette, subfamily B, bacterial), K01992 (ABC-2 type transport system permease protein) in the top 50 highest abundances of KEGG orthology (Figure 7C, Supplementary Figure S8).

Furthermore, in the clusters of orthologous groups (COGs) analysis, the findings reflected that the three most increased functional abundances of microbial community were cell wall/membrane/envelope biogenesis; post-translational modification, protein turnover, and chaperones; carbohydrate transport and metabolism, and the three most decreased in microbial community functional abundances were transcription, cell motility and signal transduction mechanisms (Supplementary Figure S9). In conclusion, these discoveries indicated that TLR4 may cause changes in gut microbiota and their associated biological function, possibly driven by specific bacterial species involved in different metabolic pathways.

## 4. Discussion

TLR4 is a type of pattern recognition receptor with a prominent role in the innate immune system, and exists in different cell types like epithelial and immune cells. It identifies bacterial LPS, a specific antigen on cell membranes of Gram-negative bacteria like *S. Typhimurium*, capable of stimulating downstream signaling cascades that activate NF-κB or the production of cytokine secretion. Nevertheless, the defense mechanisms of TLR4 overexpression in sheep against invading pathogens are still incompletely clear. In the present research, using macrophages isolated from the venous blood of TG and WT sheep, we examined the cytokines expression downstream of TLR4 under *S. Typhimurium* stimulation. Our data indicated that the production of pro-inflammatory cytokines, like IL-6, IL-1β, IL-8, and TNFα, was notably higher in TG group than the control group at different time points. These data illustrated that *S. Typhimurium* induces a stronger natural immune response in TLR4 overexpressing TG sheep. The TG group exhibited higher phagocytosis levels than the control group, in other words, more *S. Typhimurium* could be phagocytosed in the TLR4 overexpressing TG sheep. This result is coherent with other studies (Ghosh et al., 2015; Tan et al., 2015; Li et al., 2021). Consequently, TLR4 is of unparalleled importance in regulating phagocytosis and elimination of pathogens as part of the host defense mechanism.

The TLRs are associated with microbial colonization. The expression of TLR2, TLR4, and TLR5 in the jejunum of newborn ruminants was significantly correlated with the relative abundance of bacteria, which play an important role in intestinal defense during the passive immune transfer period (Zhu et al., 2021). Correlation analysis indicated that TLR4 expression was negatively correlated with the relative abundance of Bradyrhizobium and Rudaea. The genomic response to LPS in sheep blood is quite similar to that observed in human blood, supporting the use of sheep models for studies simulating human inflammatory diseases and the study of TLR-based immunomodulators (Enkhbaatar et al., 2015). Stool mainly reflects the colonic microbiota. We have utilized genetic overexpression and high-throughput sequencing techniques in sheep to confirm whether TLR4 signaling affects gut microbiota composition. Herein, careful comparison of the TLR4-overexpressing TG sheep and WT control sheep, however, demonstrated that the overexpression of TLR4 does not cause noticeable changes in intestinal microbiota richness. Intriguingly, it was unexpected that individuals in the samples of the TG group were more dispersed has suggested TLR4 can appropriately increase bacterial diversity. Furthermore, β-diversity analysis like PCA, NMDS analysis as well as Hierarchical clustering analysis demonstrated that the whole microbial community structure in the TLR4-overexpressing TG sheep significantly different from the WT group. High intestinal bacteria diversity plays central roles in maintaining intestinal stability. Studies have highlighted that early infection with *S. Typhimurium* can greatly inhibit the survival of other intestinal microbes and decrease the diversity of the gut bacterial communities (He et al., 2020). This is consistent with our results that the TG group can eliminate *S. Typhimurium* by inducing high levels of cytokine secretion, indicating that TLR4 overexpression is beneficial for improving intestinal diversity and maintaining intestinal stability.

Multiple significant differential taxonomic shifts between the TG sheep and the WT sheep were identified by 16S rRNA gene sequencing analysis. Bacteroidetes and Firmicutes are two of the most abundant bacterial phyla in host gut, and are thought to play a prominent role in regulating host carbohydrate, lipid, and bile acid metabolism (Tremaroli and Bäckhed, 2012; Tuncil et al., 2017). Our results reported that TLR4-overexpressing reduced the Firmicutes, the predominant Gram-positive phylum in the gut, which is potentially due to the reduction in the Ruminococcaceae family and Christensenellaceae family. TLR4 had a reparative effect on dextran sulfate sodium (DSS)-induced intestinal damage by upregulating IL-6 and colony stimulating factor 3 (CSF3) (Shi et al., 2019a; Shi et al., 2019b). Alterations in intestinal microbiota were involved in the regulation of inflammatory factor expression, with Ruminococcaceae negatively correlated with IL-6 and TNF-α (Wang et al., 2022). Besides, overexpression of TLR4 also resulted in an increased abundance of SCFA-producing bacteria, like Bacteroidetes, a class of gut beneficial microbes thought to enhance immune function and protect intestinal health. In patients with IBD, pro-inflammatory microbes including Ruminococcus gnavus and proteobacteria were increased, and a decrease in anti-inflammatory microbes like Bacteroidetes, were found (Dalal and Chang, 2014; Wu et al., 2020).The protective effect of other Bacteroides in animal models of colitis has also been demonstrated in previous studies (Delday et al., 2019). Additionally, at the family level, the Prevotellaceae family significantly increased in the TG group, which may be beneficial because it promotes the production of SCFAs, such as butyric acid. SCFAs stimulate the production of antimicrobial peptide substances by intestinal cells, reducing the pH in the host gut, which helps reduce the invasion and colonization of Salmonella, inhibit the development of gut inflammation, and maintain intestinal health (Fernández-Rubio et al., 2009; Kelly et al., 2015). Additionally, changes in the abundance of specific bacterial species are also deeply associated with corresponding biological functions. At the genus level, we found that, the relative abundance of Ruminococcaceae_UCG-005, Rikenellaceae_RC9_gut_group, p-251-o5, Bacteroides were up-regulated in TG group compared to WT group. Then, the abundances of Ruminococcaceae_UCG-010, [Eubacterium]_coprostanoligenes_group, Ruminococcaceae_NK4A214_group and Ruminococcaceae_UCG-013 were significantly decreased in the TLR4-overexpressing sheep. Ruminococcaceae-UCG-010 and UCG-005 have the function of carbohydrates degradation and these communities may contribute to further fermentation of feed (Kabel et al., 2011; Kim et al., 2011).

The ratio F/B is considered to measure as a marker of imbalance and dysfunction of the intestinal microbiota (Tilahun et al., 2022). Previous studies on obesity have also shown that a high-fat diet induces an increase in the F/B ratio, which is strongly associated with intestinal immune inflammation (Min et al., 2019; Li et al., 2021). In the 16 s rDNA sequencing analysis, a reduce of F/B ratio was observed in TG group, suggesting that TLR4 gene has beneficial effect in regulating gut dysbiosis. The decrease in microbial diversity is mainly manifested by an increase in abundance of facultative anaerobes, especially the phylum Proteobacteria (Shin et al., 2015). Furthermore, the increased prevalence of Proteobacteria causes dysregulation of gut microbes and is linked to diabetes, inflammation, and cancer (Shin et al., 2015). The decrease in the relative abundance of Proteobacteria observed in TG group also reflected the higher anti-inflammatory capacity and stable gut bacteria in the TG group from side. Furthermore, LEfSe analysis was applied to recognize the pivotal phylotypes of bacteria between TG and WT. Results illustrated that the principal microbiota among TG sheep were Bacteroidia, Bacteroidetes, Bacteroidales completely different from Ruminococcaceae_UGG_010, Ruminococcaceae_UGG_013, Ruminococcaceae_NK4A214_group in WT group. Overall, our findings suggested that overexpression of TLR4 affected the composition of intestinal microbiota and improved some key bacterial species abundance in sheep. Recognition of gut bacteria *via* TLR4 has a crucial role in protecting host from direct harm, and imbalanced interaction among gut bacteria and TLR4 may promote chronic inflammation and disease development, such as IBD. We provide evidence for a connection between TLR4 overexpression and the intestinal microbial structure and composition in sheep. This finding implied that TLR4 can regulate the intestinal microbial ecology.

Another function of the gut microbiome is to regulate host metabolism. Compared with the control, some metabolic pathways were enriched in TG. For instance, the abundance of the metabolism of other amino acids, glycan biosynthesis and metabolism, and energy metabolism in TG group were increased by TLR4 overexpression. Differently, the overexpression of TLR4 significantly decreased the function in a cell motility, transcription, signal transduction and endocrine system. Evidence has highlighted that colitis occurrence involves alterations in multiple genes related to signal transduction, immune cell trafficking and cell viability (Meddens et al., 2016; Neurath, 2019). These findings implied that the overexpression of TLR4 in sheep might be responsible for alleviating colitis as well as the increased intestinal barrier functions. Further studies are needed to determine the possible causal relationship.

Due to the notable differences in the microflora of sheep colonized in different institutions and under different housing and dietary conditions. Therefore, in this experiment we paid careful attention to the parental pedigree and feeding practices of sheep. Nonetheless, the limitations of this study should be considered. First, due to the small number of gene-edited large animals, the sample size of this research was restricted. However, a preliminary study with a small sample can be used as an alternative method (Al-Mekhlafi et al., 2020). These results provide evidence as to whether it is worthwhile to expand the study, and may even inform the calculation of sample size in the future study. Second, the interactions between TLR4 and the microbiome may rely on the sampling area. Subsequent experiments should focus on the tissue contents of the duodenum, jejunum, and ileum to fully illuminate the roles of TLR4 in intestinal microbiota composition and gut barrier function.

## 5. Conclusion

The present research reported that overexpression of TLR4 markedly increased sheep immunity to resist the adverse effects caused by the *S. Typhimurium* challenge. The results also indicated that the expression levels of IL-6, TNFα, IL-1β and IL-8 are promoted by TLR4 overexpression. In addition, TLR4 overexpression could affect sheep *via* regulating microbial community structure and composition. The analysis of intestinal microbiota composition revealed that overexpression of TLR4 can decrease the Firmicutes-to-Bacteroidetes ratio and the number of Proteobacteria. At the family level, TG group significantly expanded the growth of Prevotellaceae and suppressed the proliferation of Ruminococcaceae compared with WT group. These findings demonstrated that overexpression of TLR4 can maintain intestinal homeostasis by improving the relative abundance of microbial communities associated with anti-inflammation. The overexpression of TLR4 also reduced the relative abundance of several specific genera that are potentially harmful to sheep health. Thus, TLR4 could significantly alter microbial species in sheep. Prediction of metabolic function suggested that TLR4 can considerably alter the profile of metabolic pathways of bacteria. TG group showed a stronger ability to promote the metabolism of other amino acids, glycan biosynthesis and metabolism, and energy metabolism than WT group. Besides, TG group dramatically suppressed the cell motility, transcription, signal transduction and endocrine system. In summary, TLR4 has potentially beneficial impacts on the host by regulating the type and structure of the intestinal microbiota.

## Data availability statement

The datasets presented in the study are deposited in the NCBI Sequence Read Archive (https://www.ncbi.nlm.nih.gov/bioproject) under accession number PRJNA891502.

## Ethics statement

The animal study was reviewed and approved by The Animal Care and Use Committee at China Agricultural University (approval number AW81012202-1-3).

## Author contributions

X-LX: writing original draft preparation. YZ, J-LZ, and X-SZ: collecting the samples. YL, M-MC, and S-JW: editing and technical review. YL: visualization. KY and Z-XL: supervision. All authors have read and agreed to the published version of the manuscript.

## Funding

This work was supported by the National Transgenic Creature Breeding Grand Project (2016zx08008-003) and the National Key Research and Development Project of China (2021YFF1000704).

## Conflict of interest

The authors declare that the research was conducted in the absence of any commercial or financial relationships that could be construed as a potential conflict of interest.

## Publisher’s note

All claims expressed in this article are solely those of the authors and do not necessarily represent those of their affiliated organizations, or those of the publisher, the editors and the reviewers. Any product that may be evaluated in this article, or claim that may be made by its manufacturer, is not guaranteed or endorsed by the publisher.
