# Supplementary material for: Shifts in intestinal microbiota and improvement of sheep immune response to resist Salmonella infection using Toll-like receptor 4 (TLR4) overexpression
Source: Front Microbiol. 2023 Feb 15;14:1075164. doi: 10.3389/fmicb.2023.1075164 (PMC9974671; doi:10.3389/fmicb.2023.1075164)
Supplement: Supplementary file 1 [file Data_Sheet_1.docx]

**Supplementary Material:**

**
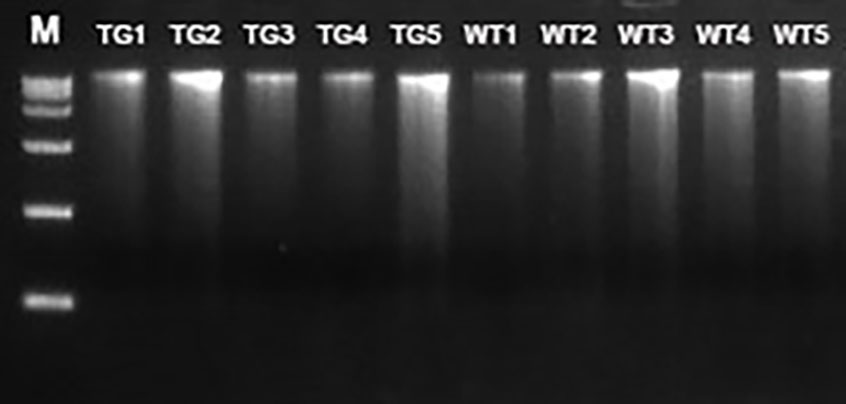
**

**Supplementary Figure S1.** The electropherogram of total microbial DNA. TG, transgenic group; WT, wild type group.

**
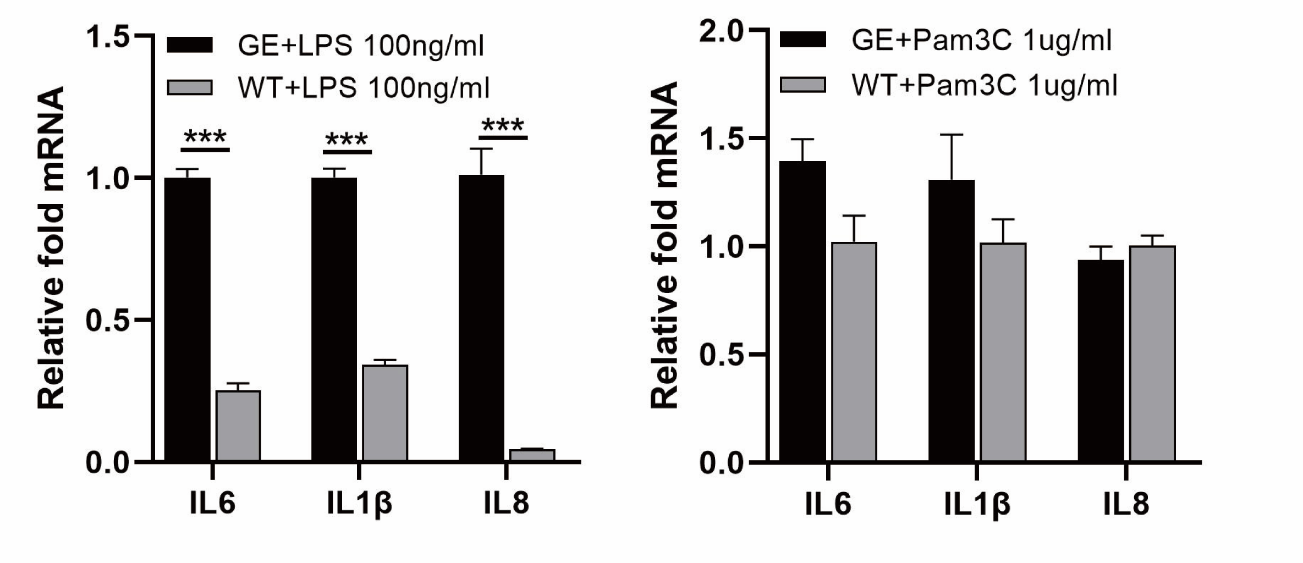
**

**Supplementary Figure S2.** TG and WT macrophages were treated with TLR4 agonist LPS (100ng/ml) or TLR2 agonist Pam3CSK4 (1 μg/ml) for 48 h. The mRNA levels of IL-6, IL-1β, and IL-8 were determined by qRT-PCR. ***P < 0.001.

**Supplementary Table S1**. Samples used in the study with total reads, final viable readings and the number of each taxon.

| **Group** | **Sample** | **Total** **reads** | **Final** **reads** | **Final reads %** | **The Number of Taxa** | | | | |
| --- | --- | --- | --- | --- | --- | --- | --- | --- | --- |
|  |  |  |  |  | **Phylum** | **Class** | **Order** | **Family** | **Genus** |
| Transgenic | TG1 | 119132 | 54194 | 45.49% | 13 | 19 | 27 | 50 | 135 |
|  | TG2 | 148092 | 67549 | 45.61% | 17 | 23 | 30 | 58 | 140 |
|  | TG3 | 141957 | 61172 | 43.09% | 14 | 20 | 26 | 54 | 134 |
|  | TG4 | 107815 | 36196 | 33.57% | 13 | 16 | 19 | 45 | 116 |
|  | TG5 | 132173 | 58813 | 44.50% | 15 | 22 | 37 | 70 | 160 |
| Wild-Type | WT1 | 129786 | 59887 | 46.14% | 14 | 19 | 25 | 50 | 131 |
|  | WT2 | 126291 | 53799 | 42.60% | 15 | 21 | 27 | 55 | 133 |
|  | WT3 | 142037 | 82238 | 57.90% | 15 | 20 | 23 | 53 | 136 |
|  | WT4 | 144558 | 62351 | 43.13% | 15 | 20 | 30 | 56 | 133 |
|  | WT5 | 125165 | 58902 | 47.06% | 14 | 18 | 22 | 50 | 126 |

**Supplementary Table S2.** AVSs and α-diversity indices of gut microbiota in each group. Data are presented as the means ± SD (n = 5). TG, transgenic group; WT, wild type group.

| Groups | ASVs | Richness index for: | | Diversity index for: | |
| --- | --- | --- | --- | --- | --- |
|  |  | Chao1 | Shannon | | Simpson |
| TG | 6164.6±453.61 | 7829.752±581.20 | 11.281±0.2306 | | 0.9986±0.0006 |
| WT | 5625.8±756.97 | 7455.052±843.86 | 11.146±0.2402 | | 0.9989±0.0003 |
| P value |  | 0.6 | 0.35 | | 0.46 |

**
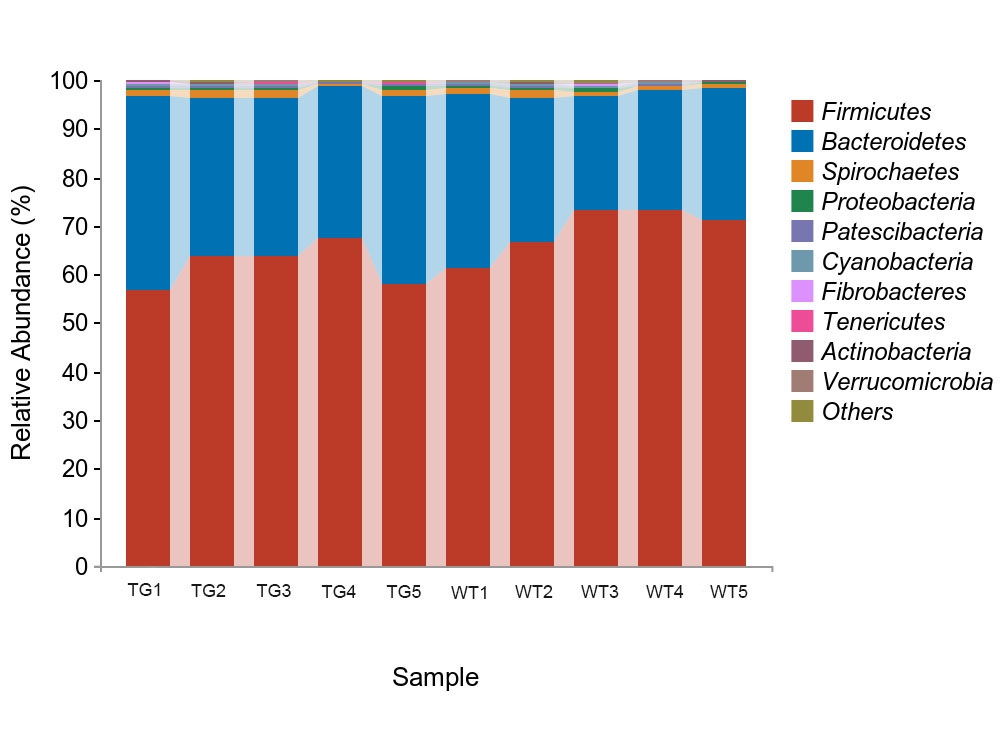
**

**Supplementary Figure S3.** The relative abundance of phylum in each group, with the Firmicutes and the Bacteroidota phyla dominating. Each color represents a phylum, and the height of the column represents the abundance of readings. Only bacterial phyla with relative abundance in the top 10 of all samples are shown. All other unassigned and classified ASVs are represented as Others/Unassigned.


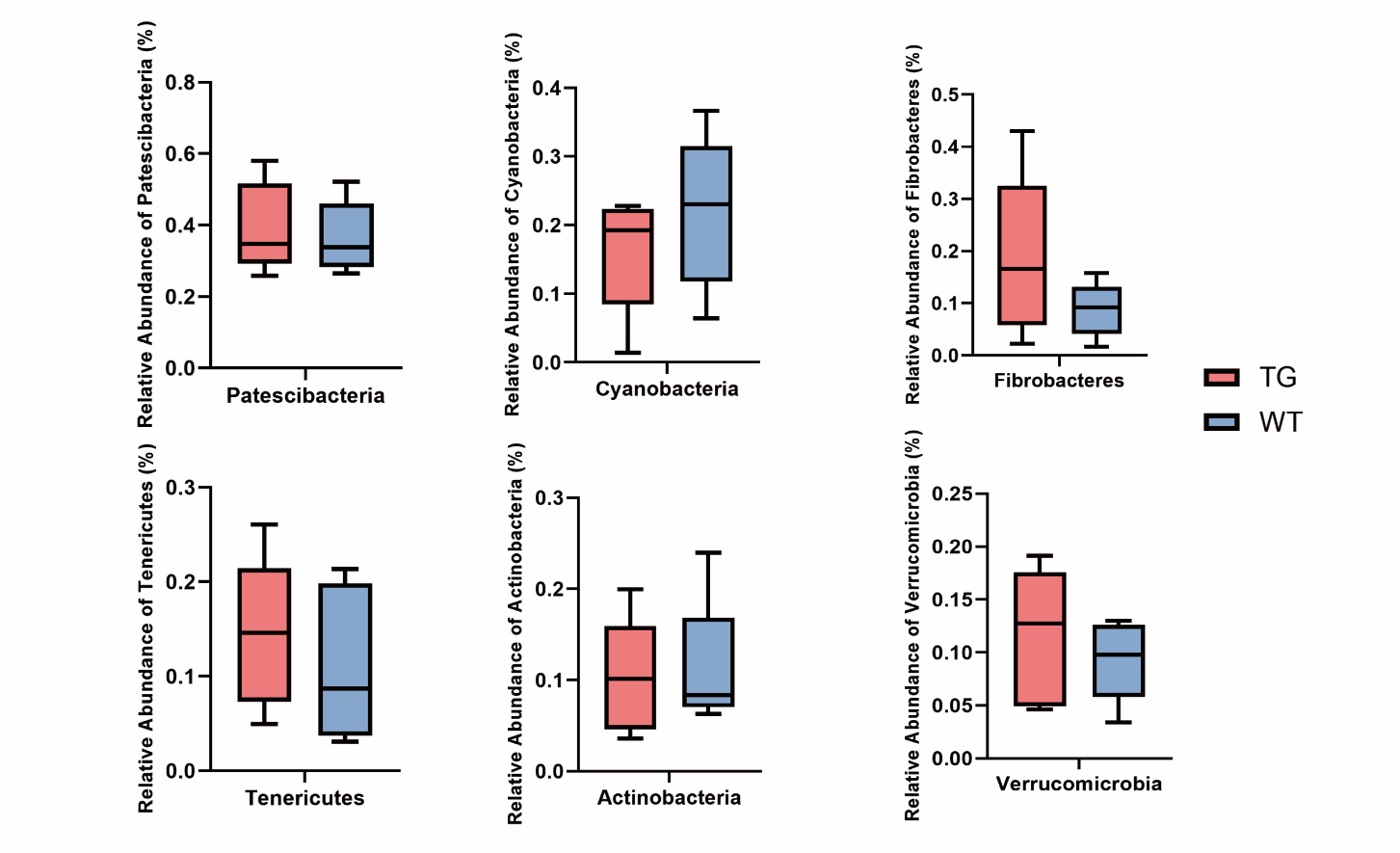


**Supplementary** **Figure S4.** Influence of TLR4 in sheep at the phylum level. The abundances of each phylum were assessed for significant differences between TG vs WT. TG, transgenic group; WT, wild type group.


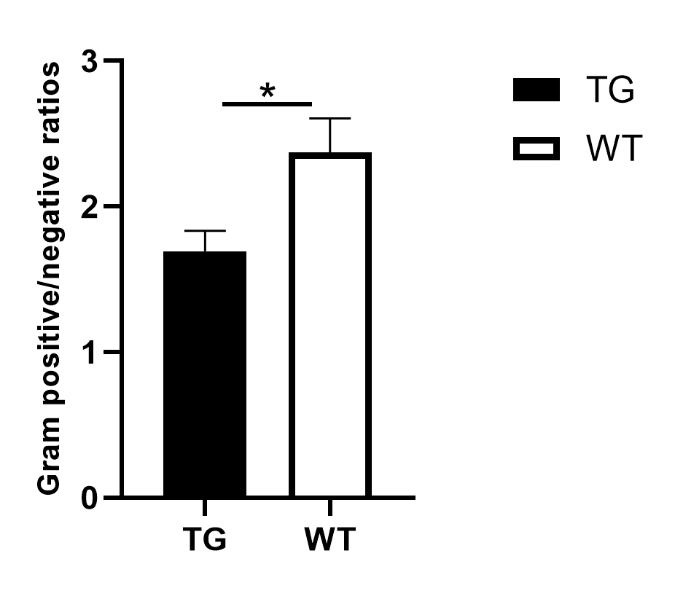


**Supplementary Figure S5.** Statistical analysis of Gram positive/negative ratios. *p < 0.05 compared with WT group. TG, transgenic group; WT, wild type group.

**Supplementary Table S3.** The relative abundance of genera higher than 0.1% in any group were shown. Data are presented as the average. *p < 0.05, **p < 0.01 vs WT. Red arrows represent increasing, blue arrows represent decrease of relative abundance vs control group. TG, transgenic group; WT, wild type group.

| **Genus name** | **TG**  **Average (%)** | **WT**  **Average (%)** | **P-value** | **Variation tendency** |
| --- | --- | --- | --- | --- |
| *Ruminococcaceae_UCG-005* | 12.272 | 12.203 | 0.954 | ↑ |
| *Christensenellaceae_R-7_group* | 10.316 | 11.198 | 0.239 | ↓ |
| *Rikenellaceae_RC9_gut_group* | 10.533 | 10.057 | 0.456 | ↑ |
| *p-251-o5* | 9.007 | 4.626 | 0.067 | ↑ |
| *Ruminococcaceae_UCG-010* | 4.613 | 6.778 | 0.014 | ↓* |
| *[Eubacterium]_coprostanoligenes group* | 3.446 | 4.604 | 0.030 | ↓* |
| *Bacteroides* | 2.630 | 2.505 | 0.648 | ↑ |
| *Ruminococcaceae_UCG-013* | 2.120 | 2.863 | 0.047 | ↓* |
| *Ruminococcus_1* | 2.207 | 2.606 | 0.474 | ↓ |
| *Ruminococcaceae_NK4A214_group* | 1.966 | 2.669 | 0.032 | ↓* |
| *Prevotellaceae_UCG-003* | 2.567 | 1.564 | 0.042 | ↑* |
| *Ruminococcaceae_UCG-014* | 1.468 | 2.089 | 0.014 | ↓* |
| *Family_XIII_AD3011_group* | 1.542 | 1.476 | 0.708 | ↑ |
| *Ruminococcaceae_UCG-002* | 1.219 | 1.699 | 0.052 | ↓ |
| *Bacteroidales_UCG-001* | 1.457 | 1.422 | 0.938 | ↑ |
| *Alistipes* | 1.219 | 1.264 | 0.813 | ↓ |
| *Treponema_2* | 1.342 | 1.077 | 0.383 | ↑ |
| *Prevotellaceae_UCG-004* | 1.160 | 0.970 | 0.236 | ↑ |
| *F082* | 0.868 | 0.875 | 0.953 | ↓ |
| *Phascolarctobacterium* | 0.693 | 0.884 | 0.017 | ↓* |
| *Ruminococcaceae_UCG-009* | 0.612 | 0.817 | 0.050 | ↓* |
| *dgA-11_gut_group* | 0.469 | 0.691 | 0.021 | ↓* |
| *M2PB4-65_termite_group* | 0.520 | 0.636 | 0.449 | ↓ |
| *Oscillibacter* | 0.432 | 0.621 | 0.062 | ↓ |
| *[Eubacterium]_nodatum_group* | 0.652 | 0.400 | 0.086 | ↑ |
| *Bacteroidales_RF16_group* | 0.557 | 0.481 | 0.460 | ↑ |
| *Candidatus_Soleaferrea* | 0.419 | 0.502 | 0.119 | ↓ |
| *[Eubacterium]_oxidoreducens_group* | 0.487 | 0.331 | 0.115 | ↑ |
| *Romboutsia* | 0.332 | 0.457 | 0.406 | ↓ |
| *Candidatus_Saccharimonas* | 0.392 | 0.365 | 0.722 | ↑ |
| *Ruminiclostridium* | 0.359 | 0.380 | 0.859 | ↓ |
| *Lachnoclostridium_10* | 0.385 | 0.346 | 0.624 | ↑ |
| *Ruminococcaceae_UCG-004* | 0.281 | 0.350 | 0.019 | ↓* |
| *Dorea* | 0.368 | 0.239 | 0.035 | ↑* |
| *Lachnospiraceae_AC2044_group* | 0.289 | 0.301 | 0.797 | ↓ |
| *Ruminococcus_2* | 0.277 | 0.292 | 0.646 | ↓ |
| *p-2534-18B5_gut_group* | 0.350 | 0.204 | 0.244 | ↑ |
| *Ruminiclostridium_1* | 0.228 | 0.285 | 0.103 | ↓ |
| *Ruminiclostridium_6* | 0.261 | 0.252 | 0.853 | ↑ |
| *Ruminiclostridium_9* | 0.221 | 0.282 | 0.117 | ↓ |
| *Flavonifractor* | 0.232 | 0.269 | 0.423 | ↓ |
| *Clostridiales_vadinBB60_group* | 0.160 | 0.300 | 0.007 | ↓** |
| *Prevotellaceae_UCG-001* | 0.257 | 0.175 | 0.214 | ↑ |
| *Mailhella* | 0.167 | 0.235 | 0.336 | ↓ |
| *Prevotella_1* | 0.220 | 0.181 | 0.531 | ↑ |
| *Gastranaerophilales* | 0.157 | 0.219 | 0.346 | ↓ |
| *Lachnospiraceae_NC2004_group* | 0.206 | 0.149 | 0.355 | ↑ |
| *Papillibacter* | 0.143 | 0.210 | 0.053 | ↓ |
| *Agathobacter* | 0.210 | 0.125 | 0.233 | ↑ |
| *Paeniclostridium* | 0.090 | 0.242 | 0.156 | ↓ |


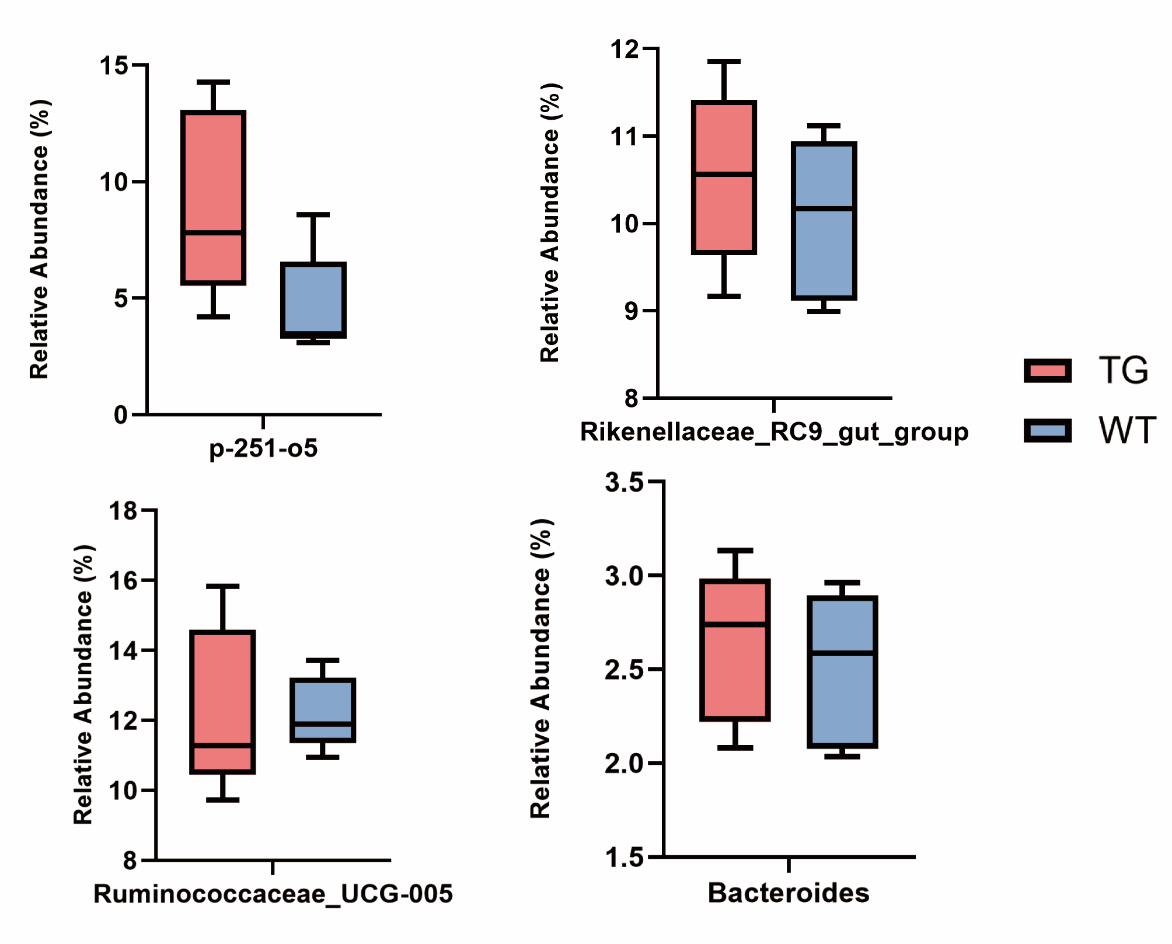


**Supplementary Figure S6.** Influence of genotype in sheep at the genus level. TG, transgenic group; WT, wild type group.


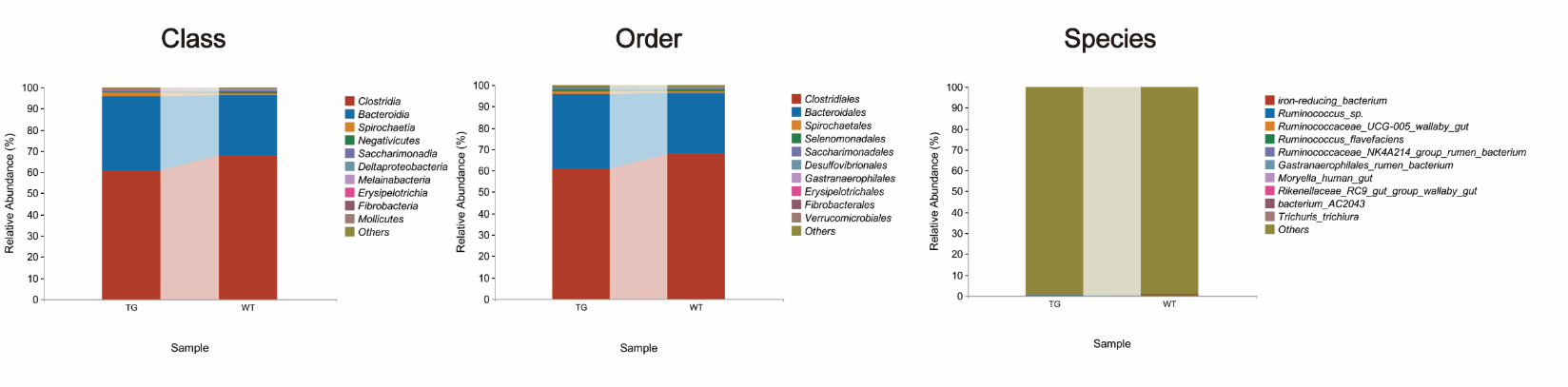


**Supplementary Figure S7.** Composition analysis of gut microbiota at a class, order and species level. TG, transgenic group; WT, wild type group.


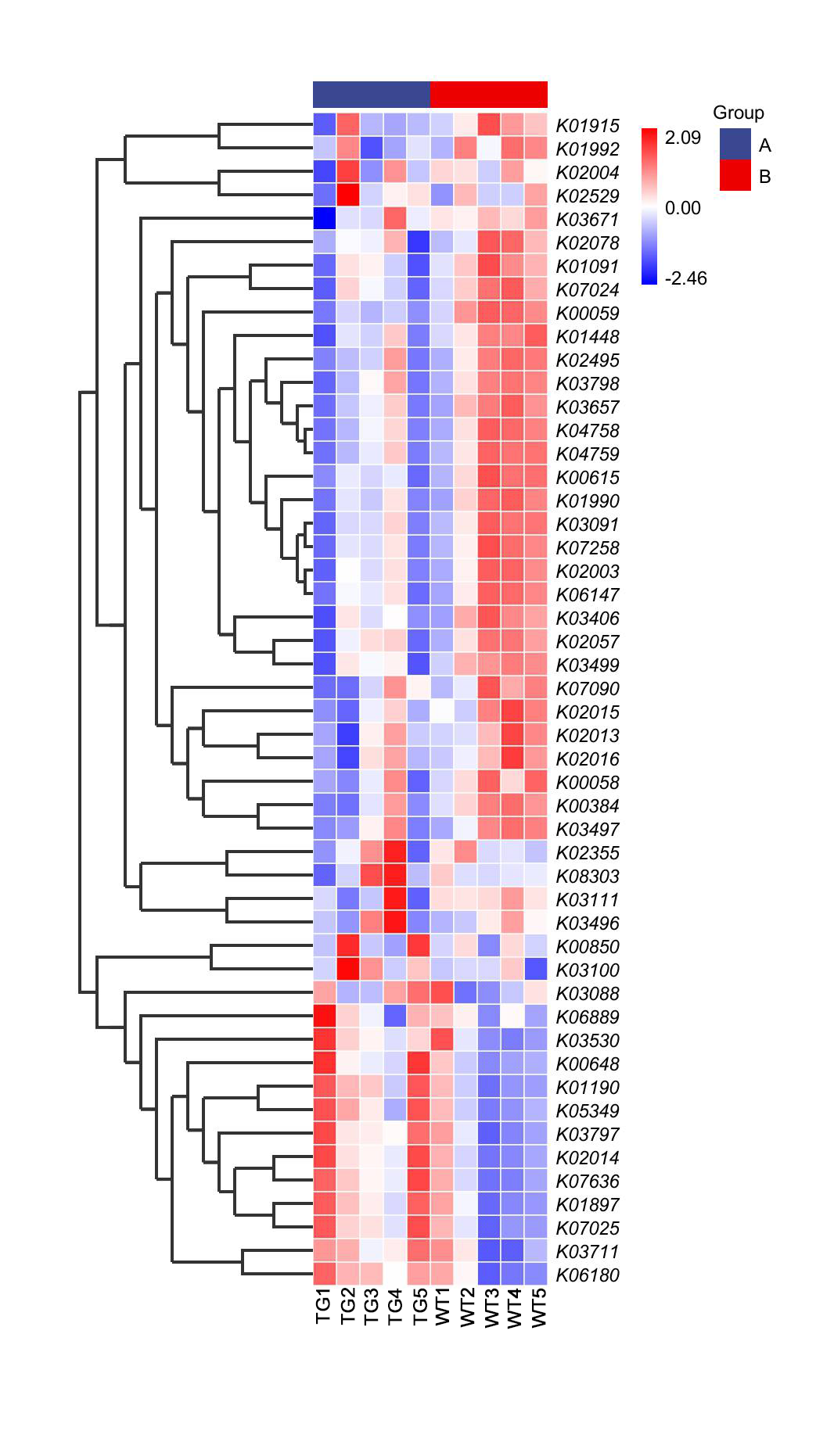


**Supplementary Figure S8.** Prediction of microbiome function based on Kyoto Encyclopedia of Genes and Genomes (KEGG) database.


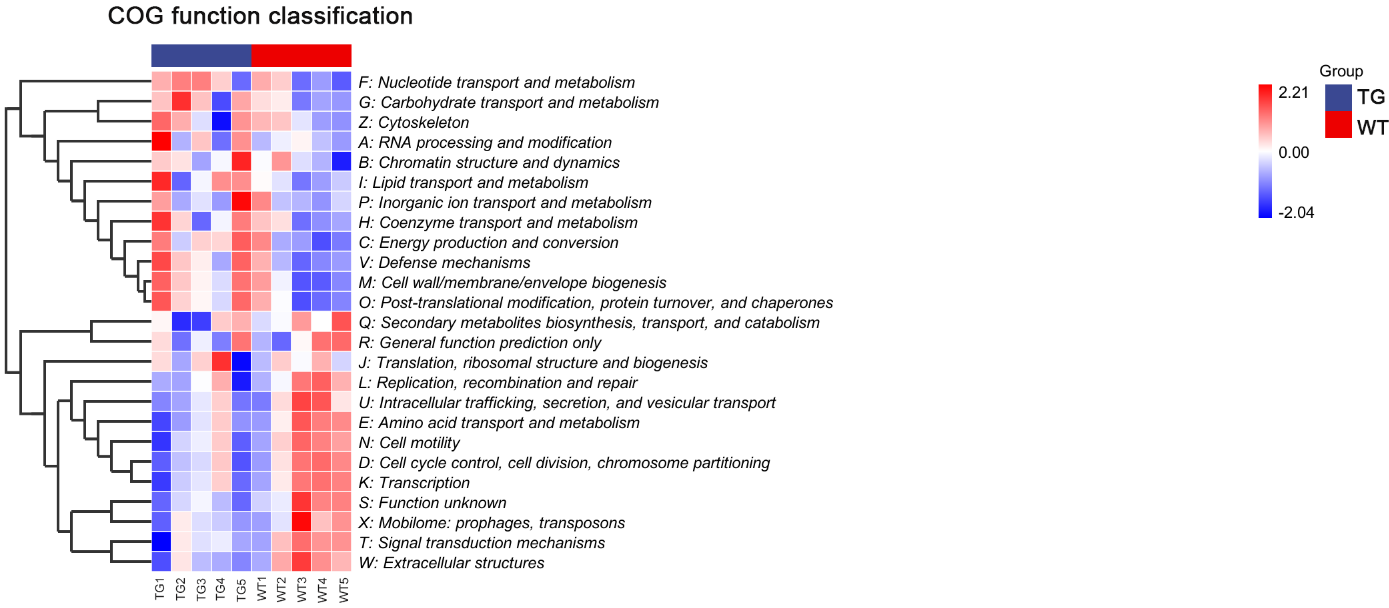


**Supplementary Figure S9.** The clusters of orthologous groups (COGs) analysis displayed the top increased and the top decreased functional abundances of microbial community. TG, transgenic group; WT, wild type group.
